# Biomarkers for determining the prognosis in chronic myelogenous leukemia

**DOI:** 10.1186/1756-8722-6-54

**Published:** 2013-07-19

**Authors:** Kendra Sweet, Ling Zhang, Javier Pinilla-Ibarz

**Affiliations:** 1 H. Lee Moffitt Cancer Center & Research Institute, 13131 Magnolia Drive 3 East, Room 3056H, Tampa, FL 33612, USA

**Keywords:** Chronic myelogenous leukemia, BCR-ABL1, Philadelphia chromosome, Kinase domain mutation, Imatinib resistance, Tyrosine kinase inhibitors, Prognosis

## Abstract

The introduction of BCR-ABL1 tyrosine kinase inhibitors (TKIs) for treatment of chronic myelogenous leukemia in chronic phase (CML-CP) has revolutionized therapy, altering the outcome from one of shortened life expectancy to long-term survival. With over 10 years of long-term treatment with imatinib and several years of experience with the next generation of TKIs, including nilotinib, dasatinib, bosutinib, and ponatinib, it is becoming clear that many clinical parameters have great impact on the prognosis of patients with CML. Emerging novel gene expression profiling and molecular techniques also provide new insights into CML pathogenesis and have identified potential prognostic markers and therapeutic targets. This review presents the supporting data and discusses how certain clinical characteristics at diagnosis, the depth of early response, the presence of certain kinase domain mutations, and additional molecular changes serve as prognostic factors that may guide individualized treatment decisions for patients with CML-CP.

## Introduction

Chronic myelogenous leukemia (CML) is a clonal myeloproliferative neoplasm caused by constitutive activation of the BCR-ABL1 tyrosine kinase, a result of the t(9;22)(q34;q11) translocation designated the Philadelphia (Ph) chromosome [1]. Treatment targeting this kinase has proven to be highly successful. The introduction of imatinib, the first BCR-ABL1 tyrosine kinase inhibitor (TKI), overturned the treatment paradigm for CML [2,3] by extending survival in patients who maintain durable response to continued imatinib therapy [4,5].

Although imatinib represented a major therapeutic advance, the need for additional treatments was recognized and addressed by the development of the more potent BCR-ABL1 TKIs nilotinib, dasatinib, bosutinib, and ponatinib (Table 1). Nilotinib and dasatinib were approved for first-line treatment of Ph + CML in chronic phase (CP) based on results of separate phase 3 studies indicating superior cytogenetic and molecular response rates compared with imatinib [6-9]. Three- and 4-year data from both studies continue to demonstrate significantly greater response rates for nilotinib and dasatinib versus imatinib [10-12].

**Table 1:** US FDA-Approved BCR-ABL1 TKIs

**Name**	**Year approved**	**Indications in CML**
Imatinib	2001	CP, AP, or BC after failure of interferon therapy
	2003	Newly diagnosed CP
Dasatinib	2006	CP, AP, or BC after resistance to or intolerance of imatinib
	2010	Newly diagnosed CP
Nilotinib	2007	CP or AP after resistance to or intolerance of imatinib
	2010	Newly diagnosed CP
Bosutinib	2012	CP, AP, or BC after resistance to or intolerance of prior therapy
Ponatinib	2012	CP, AP, or BC after resistance to or intolerance of prior TKI therapy

*AP* accelerated phase, *BC* blast crisis, *CML* chronic myelogenous leukemia, *CP* chronic phase, *FDA* Food and Drug Administration, *TKI* tyrosine kinase inhibitor, *US* United States.

With 3 TKIs currently available for front line therapy and 2 more TKIs for second- and third-line therapy, questions arise regarding which agent should be used, and when, to optimize long-term outcomes. Numerous patient- and drug-related factors, in addition to financial considerations, contribute to decisions on treatment selection. Emerging evidence suggests that certain clinical characteristics at diagnosis, the depth of early response, the presence of certain kinase domain (KD) mutations, and additional molecular changes may impact the prognosis of CML patients. This review examines various prognostic factors in CML and explores the practical utility of these prognostic factors in guiding treatment decisions for patients with CML-CP both now and in the future.

### Prognostic indicators at diagnosis

*Prognostic scoring systems.* CML prognostic scoring systems stratify patients into risk groups based on patient- and disease-related characteristics at diagnosis. Until recently, there were 2 widely used scoring systems, Sokal and Hasford (Table 2). Introduced in 1984, the Sokal score could classify patients treated with standard chemotherapy (busulfan or hydroxyurea) into 3 risk groups, each with significantly different predicted long-term survival [13]. With wider use of interferon-α for early-stage CML, the Sokal score lost prognostic utility and the Hasford score was developed [14].

**Table 2:** **Sokal, Hasford, and EUTOS Systems**[13-15]

	**Sokal**	**Hasford (EURO)**	**EUTOS**
Year introduced	1984	1998	2011
Predominant treatment modality	Conventional chemotherapy	Interferon-α–based regimens	Imatinib
Factors	•Age	•Age	•Spleen size
	•Spleen size	•Spleen size	•Basophil count
	•Platelet count	•Platelet count	
	•Percentage of blasts	•Percentage of blasts	
		•Percentage of basophils	
		•Percentage of eosinophils	
Calculation of score	Exp 0.0116 (age – 43) + 0 .0345 (spleen size [cm below costal margin] – 7.5 cm) + 0.188 [(platelet count/700)^2^ – 0.563] + 0.0887 (% blasts in blood – 2.1)	(0.6666 × age [0 when age <50 years; 1, otherwise] + 0.0420 × spleen size [cm below costal margin] + 0.0584 × blasts [%] + 0.0413 × eosinophils [%] + 0.2039 × basophils [0 when basophils <3%; 1, otherwise] + 1.0956 × platelet count [0 when platelets <1500 × 10^9^/L; 1, otherwise]) × 1000	7 × basophils + 4 × spleen size
Risk groups^a^	•High: score > 1.2	•High: score >1480	•High: score > 87
	•Intermediate: score 0.8-1.2	•Intermediate: score >780 and ≤1480	•Low: score ≤ 87
	•Low: score < 0.8	•Low: score ≤ 780	

^a^An intermediate risk category is not defined for EUTOS.

In 2011, the European Treatment and Outcome Study (EUTOS) score was introduced to reflect current standard use of first-line imatinib (Table 2) [15]. In a comparative evaluation, the EUTOS score was better than Sokal or Hasford scores at predicting complete cytogenetic response (CCyR) at 18 months (positive predictive value, 34%; sensitivity, 23%; specificity, 92%) and progression-free survival (PFS) at 5 years (sensitivity, 16%; specificity, 91%) [15]. In an analysis of the German CML-Study IV, EUTOS classification (high versus low risk) significantly correlated with achievement of major molecular response (MMR) and complete molecular response (CMR) [16].

Not all groups have been able to validate the EUTOS score, however. An analysis from Hammersmith Hospital of 277 imatinib-treated patients found that Sokal score, but not EUTOS score, predicted overall survival (OS), PFS, CCyR, and MMR [17]. The MD Anderson Cancer Center group showed that EUTOS score was not successful at predicting outcomes (MMR, transformation-free survival, event-free survival [EFS], or OS) in an analysis of CML-CP patients treated with imatinib (n = 279), nilotinib (n = 98), or dasatinib (n = 88) [18]. Disparate conclusions about the utility of the EUTOS score may be due to differences in patient populations evaluated or its inapplicability to patients receiving first-line nilotinib or dasatinib.

The applicability of Sokal and Hasford scores for patients receiving newer TKIs is also unclear. In the Evaluating Nilotinib Efficacy and Safety in Clinical Trials–Newly Diagnosed Patients (ENESTnd) study, nilotinib-treated patients had higher rates of MMR, CMR, and CCyR by 24 months than imatinib-treated patients, regardless of Sokal risk category [10]. Similarly, the Dasatinib versus Imatinib Study in Treatment-Naive CML Patients (DASISION) study found higher 24-month MMR rates with dasatinib versus imatinib across Hasford risk categories [19]. Interestingly, in both the ENESTnd and DASISION studies, patients who progressed in the imatinib arms were categorized as intermediate or high risk patients per the respective scoring systems. In the Bosutinib Efficacy and Safety in Newly Diagnosed CML (BELA) study, patients on bosutinib had higher rates of 12-month MMR than patients on imatinib, regardless of Sokal risk category [20].

These findings suggest that the parameters used in these prognostic scoring systems are limited, mostly clinically oriented, and not directly related to genetic or molecular indicators. Nevertheless, because high-risk patients in the ENESTnd and DASISION studies experienced less disease progression on nilotinib and dasatinib, respectively, than on imatinib, NCCN Guidelines recommend determination of Sokal or Hasford risk status as part of the initial workup and the use of nilotinib or dasatinib in high-risk patients [5]. Further validation of the EUTOS score will also be necessary before it is used in routine practice.

*“Real-world” prognostic factors.* Most data regarding imatinib use are from clinical studies; data from real-world settings are sparse. A recent study investigated prognostic factors associated with achievement of complete hematologic response, CCyR, MMR, and CMR in 1063 patients on first-line imatinib treatment who had not participated in clinical studies [21]. Low Sokal risk score, age <45 years, and African-American ethnicity were associated with better outcomes [21]. How widely considered these specific prognostic factors are in routine risk assessment and whether they are applicable to nilotinib- or dasatinib-treated patients are unknown.

*Prognostic impact of additional cytogenetic aberrations (ACAs).* ACAs are documented in 10%-15% of newly diagnosed patients before TKI treatment [22]. In a retrospective analysis of the German CML Study IV, patients with “major route” ACAs, including an additional Ph chromosome, trisomy 8, isochromosome 17q, and trisomy 19 [23,24], at diagnosis had significantly longer median times to CCyR and MMR, and reduced 5-year PFS and OS compared to patients without ACAs [24]. Other studies have demonstrated that CML-CP patients who developed ACAs during imatinib treatment had significantly worse outcomes than patients who did not [25]. The emergence of ACAs during treatment signifies clonal evolution and, by definition, disease transformation to accelerated phase/blast crisis (AP/BC) [26,27]. Current guidelines recommend bone marrow cytogenetic testing at diagnosis, when patients respond inadequately to first-line treatment, and when patients show increasing disease burden [5]. The presence of ACAs, especially major-route abnormalities, at diagnosis may indicate high risk for poor prognosis and may justify the use of a next-generation TKI over imatinib as initial therapy.

*Variant translocations.* Nearly all patients with CML have a *BCR-ABL1* fusion gene from the t(9;22)(q34;q11) translocation. Approximately 5%-10% of patients, however, have more complex rearrangements involving chromosomes 9, 22, and one or more additional chromosomes [28]. Many variants have been identified, highlighting the genetic heterogeneity of these patients [29-32]. The prognostic significance of variant translocations remains controversial [30,31,33-35], however, and requires further study. Thus, this parameter has not yet been widely applied in treatment decision-making for patients with CML.

*BCR-ABL1 transcript: e13a2 (b2a2) versus e14a2 (b3a2).* Most mRNAs transcribed from *BCR-ABL1* have either an e13a2 or e14a2 junction. Although both mRNAs encode the p210 product of *BCR-ABL1*[36,37], the e14a2 transcript positively correlates with response. In one study, patients with the e14a2 transcript achieved higher rates of CCyR at 12 months and achieved CCyR more rapidly than patients with the e13a2 transcript [37]. In another study, MMR and MR^4^ (*BCR-ABL1* ≤ 0.01%) were achieved more rapidly by patients with the e14a2 versus the e13a2 transcript [38]. At present, this parameter is not widely used in routine practice, in large part because many commercial molecular testing laboratories do not report the type of *BCR-ABL1* transcript. Furthermore, although these data are suggestive, further investigation will be necessary to conclusively determine the prognostic utility of *BCR-ABL1* transcript type.

*Pharmacokinetics.* The organic cation transporter-1 (OCT-1) is the major transporter of imatinib into CML cells [39]. OCT-1 activity, which reflects the degree of imatinib influx, can predict long-term risk of resistance and transformation [40]. Patients with high OCT-1 activity were significantly more likely to achieve MMR by 5 years, and have significantly higher OS and EFS rates and lower KD mutation rates than patients with low activity. Although OCT-1 activity may predict imatinib failure, it is unlikely to affect the response to nilotinib, dasatinib, or ponatinib, as influx of these drugs does not rely on OCT proteins [39,41,42]. The effect of OCT-1 acitivity on bosutinib influx is unknown [43]. The measurement of OCT-1 activity level is currently limited to clinical research and is not yet considered routine practice.

Imatinib plasma levels may correlate with treatment response. In one study, mean imatinib trough levels were significantly higher in patients who achieved CCyR and MMR than in patients who did not (1123 versus 694 ng/mL, *P* = 0.03) [44]. An exploratory analysis from the International Randomized Study of Interferon and STI571 (IRIS) found that imatinib trough levels predicted rates of CCyR, MMR, and EFS [45]. Some subsequent studies have confirmed these findings; others have not [45-48]. Because the clinical benefit of treatment change based on TKI plasma levels is unproven, current guidelines do not recommend routine monitoring of this sort [5].

### Prognosis based on early treatment response: landmark analyses

Landmark analyses assess treatment responses at fixed timepoints and correlate them with future endpoints or clinical outcomes. Landmark analysis of the IRIS study found that molecular responses at 6, 12, and 18 months predicted outcomes at 7 years [49]. The 7-year PFS rate was 99% for patients who attained MMR by 12 or 18 months, compared with 90% for patients who did not. Rates of 7-year EFS were 95%, 86%, and 65% in patients who achieved MMR, *BCR-ABL1* >0.1% to ≤1.0% per the international scale (IS), and *BCR-ABL1*^IS^ >1.0%, respectively, at 18 months [49]. These data suggested that response kinetics are important, with rapid and deep molecular responses predicting excellent long-term outcomes.

**Figure 1:**
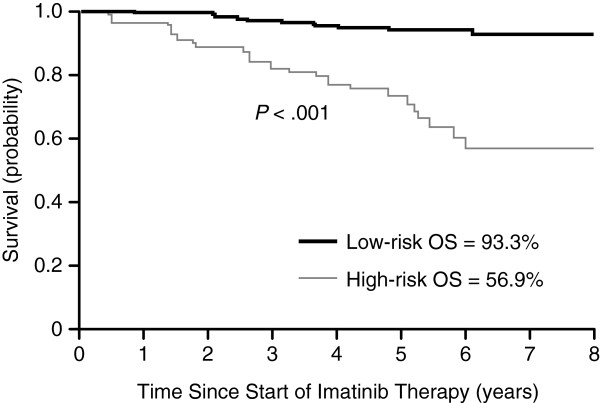
**Transcript levels predict survival outcomes.** Eight-year probability of OS for patients stratified by risk group defined by transcript levels at 3 months (high-risk *BCR-ABL1*/*ABL1* ratio > 9.84% [n = 68; gray line]; low-risk *BCR-ABL1*/*ABL1* ≤ 9.84% [n = 211; black line]). From Marin et al. [51] (reproduced with permission).

In particular, molecular response to TKI therapy at 3 months predicts future molecular response and long-term survival outcomes, including OS (Table 3) [50,51]. In one landmark analysis of imatinib-treated patients, *BCR-ABL1* transcript level at 3, 6, and 12 months predicted the 8-year rates of CCyR, MMR, CMR, OS, PFS, and EFS, but only *BCR-ABL1* transcript level at 3 months was found to be an independent predictor of 8-year survival outcomes (Figure 1) [51].

**Table 3:** Summary of 3-month landmark analyses of selected clinical studies of first-line TKI therapy

**TKI**	**Study**	**Parameter**	***BCR-ABL1*% (IS) at 3 months**		***P *value**
Imatinib	Hammersmith Hospital [51]		≤9.84% (n = 211)	>9.84% (n = 68)	
		8-year OS	93.3%	56.9%	<0.001
			≤9.54% (n = 208)	>9.54% (n = 71)	
		8-year PFS	92.8%	57.0%	<0.001
			≤8.58% (n = 169)	>8.58% (n = 79)	
		8-year CCyR	99.4%	21.7%	<0.001
			≤2.81% (n = 141)	>2.81% (n = 137)	
		8-year MMR	82.5%	21.1%	<0.001
	German CML Study IV [50]		>1-10% (n = 281)	>10% (n = 189)	
		5-year OS	92%	87%	0.037
			>1-10% (n = 283)	>10% (n = 191)	
		5-year PFS	94%	87%	0.012
	ENESTnd^a^[52]		≤10% (n = 176)	>10% (n = 88)	
		MMR by 2 years	58%	21%	NR
		PFS at 3 years	97.7%	83.8%	
		OS at 3 years	98.9%	84.8%	
	DASISION^a^[9]		≤10% (n = 154)	>10% (n = 85)	
		AP/BC by 3 years	2.6%	12.9%	NR
		PFS at 3 years	95.9%	75.3%	<0.0001
		OS at 3 years	96.0%	88.0%	0.0036
	BELA^a^[53]		≤10% (n = 146)	>10% (n =77)	
		MMR by 24 months	69%	17%	<0.001
		CCyR by 12 months	95%	65%	<0.001
		OS at 24 months	99%	95%	NS
Nilotinib	ENESTnd [52]		≤10% (n = 234)	>10% (n = 24)	
		MMR by 2 years	80%	29%	NR
		PFS at 3 years	95.9%	82.9%	
		OS at 3 years	97.6%	86.7%	
Dasatinib	DASISION [9]		≤10% (n = 198)	>10% (n = 37)	
		AP/BC by 3 years	3.0%	13.5%	NR
		PFS at 3 years	93.1%	68.2%	0.0003
		OS at 3 years	95.9%	85.9%	0.0348
Bosutinib	BELA [53]		≤10% (n = 179)	>10% (n =29)	
		MMR by 24 months	74%	21%	<0.001
		CCyR by 12 months	96%	48%	<0.001
		OS at 24 months	99%	88%	0.004

^a^Data for the imatinib arm of the study.

*AP/BC* accelerated phase/blast crisis, *CCyR* complete cytogenetic response, *MMR* major molecular reponse, *NR* not reported, *NS* not significant, *OS* overall survival, *PFS* progression-free survival.

Other studies have found evidence supporting the prognostic significance of achieving molecular response at 3 months in patients treated with imatinib, nilotinib, dasatinib, or bosutinib (Table 3). Notably, early responses in these studies were predictive of positive long-term outcomes, irrespective of TKI received. Furthermore, the newer TKIs produced faster molecular responses than imatinib, which is consistent with data from the ENESTnd, DASISION, and BELA trials [7,9,20,22,52-55].

Based on strong evidence that rapid, deep molecular responses predict favorable long-term outcomes, the National Comprehensive Cancer Network Clinical Practice Guidelines in Oncology (version 4.2013) have incorporated, for the first time, a molecular response goal of *BCR-ABL1* ≤10% at 3 months [5]. The newer TKIs are superior to imatinib in eliciting rapid response, but longer-term experience with the newer TKIs in the front-line setting will be necessary to help guide treatment decisions. Furthermore, the NCCN Guidelines recommend that patients not reaching the goal of *BCR-ABL1* ≤10% at 3 months be considered for a treatment change—in other words, replacing their current therapy with an alternative TKI. At present, however, the long-term clinical benefit of an early switch in treatment remains under investigation.

### Predictive utility of mutational analysis

Point mutations in the *BCR-ABL1* KD are a key mechanism of resistance [56]. Most KD mutations acquired during imatinib treatment confer resistance and predict poor prognosis. The presence of KD mutations often portends disesase progression, especially when patients do not respond to second-line TKI therapy. Thus, the NCCN Guidelines recommend that mutational analysis be done when patients show inadequate initial response to TKI therapy, or when there is evidence of relapse or disease progression [5].

Regarding primary resistance, attempts to identify KD mutations at diagnosis have been unsuccessful to date because such TKI-resistant subclones exist at levels too low to be detectable using conventional methodologies [57,58]. Although the presence of mutations at low levels may or may not predict poor prognosis [59], an expert panel convened by the European LeukemiaNet has nevertheless recommended that newly diagnosed patients with advanced disease be tested for mutations [56].

A more-sensitive technique for detecting TKI-resistant clones has been developed: a multiplexed mass spectrometry assay [60]. This method has been used to detect nilotinib- and/or dasatinib-resistant mutations in patients with imatinib resistance, including clinically relevant mutations that were not detectable by direct sequencing [60]. Validation of this technique for detecting low-level mutations in patients at diagnosis would help guide the most appropriate front-line TKI and, in the case of a multi–drug-resistant mutation, consideration of appropriate therapy.

### Advances in identifying molecular markers of progression

Identification of potentially important disease mediators is an active area of research that employs techniques such as gene microarray profiling and proteomic analysis. An in-depth exploration of the potential biological significance of the molecules identified is beyond the scope of this review. They are nevertheless mentioned to provide a glimpse of what the future may bring in terms of potential prognostic indicators and therapeutic targets.

Microarray profiling has been used to determine whether the specific gene expression at diagnosis can predict the response to TKI treatment [61]. Analysis of CD34+ cells from newly diagnosed, treatment-naive patients with CML-CP has revealed a 75-transcript signature (50 upregulated and 25 downregulated transcripts) that predicted major cytogenetic response at 12 months with an overall accuracy of 87% — exceeding the predictive ability of the Sokal score [62]. Notably, 62% of the upregulated transcripts were potential targets of the WNT/β-catenin pathway, which is activated during BC. Thus, gene expression profiling at diagnosis may be useful for identifying poor-prognosis patients and for elucidating the biological basis of CML disease progression [62].

Radich et al. used gene microarrays to explore what changes in gene expression are associated with progression to AP/BC. Their group identified components of the WNT/β-catenin pathway and alternative kinase pathways, transcription factors *JUN-B* and *FOS*, and the marker PRAME as being associated with advanced CML [63]. Further research identified a 6-gene signature (*NOB1, DDX47, IGSF2, LTB4R, SCARB1*, and *SLC25A3)* that discriminated between patients in CP and those in BC [64]. A second 6-gene signature (*RALGDS, LASP1, G6PD, ADRBK1, LRPPRC,* and *PSMA1*) has been identified that can predict relapse in patients with CML-CP [65].

Proteomic analysis has identified a group of proteins expressed at low levels in CML-CP but at high levels in CML-BC: HSP90, RB, AIF, PP2A, BCL2, XIAP, SMAD1, SSBP2α, PARP, GAB2, and TRIM24. A reverse pattern, high levels in CML-CP but low levels in BC, has been found for PKC.p664, AKTpT308, actin, p70S6Kp, Rac1.2.3, PDK1p, MEK, and CDK4 [66].

Other approaches have targeted known genes important in proliferation, differentiation, or cell survival. One study found increased expression of CaMKIIγ and HSP70, but decreased expression of HSP90, in patients with KD mutations compared to those without. Using this expression pattern, the likelihood of TKI-treated patients harboring resistant KD mutations could be predicted with a specificity of 82% [67]. These and other efforts are underway to further relate molecular markers to progression, with the hope that a greater understanding of their expression will help guide optimal treatment decisions.

## Conclusions

With the approval and availability of 3 front line TKIs and 2 additional TKIs after failure of previous ones for the treatment of CML, prognostic indicators to guide treatment selection have become increasingly important. Given the early state of research, there are few factors that can guide TKI selection. However, knowledge regarding diagnostic systems and genetic, molecular, and pharmacokinetic markers is advancing rapidly. Ongoing work continues to illuminate the pathways and genes that could serve both as prognostic indicators and as targets for drug development.

Convincing data are emerging that early and deep molecular responses are associated with excellent long-term outcomes. Patients who do not attain such responses have poorer prognosis and may benefit from an early change in treatment. Collectively, this abundance of knowledge suggests that we should someday be able to pinpoint the optimal treatment for each individual patient.

## Abbreviations

ABL1: Abelson 1 gene; ACA: Additional cytogenetic aberration; ADRBK1: Adrenergic, beta, receptor kinase 1; AIF: Apoptosis-inducing factor; AKT: Derived from “thymoma arising in the Ak strain of mice”; AP: Accelerated phase; BC: Blast crisis; BCL2: B-cell chronic lymphocytic leukemia/lymphoma 2; BCR: Breakpoint cluster region; BELA: Bosutinib Efficacy and Safety in Newly Diagnosed CML; CCyR: Complete cytogenetic response; CDK4: Cyclin-dependent kinase 4; CML: Chronic myelogenous leukemia; CMR: Complete molecular response; CP: Chronic phase; DASISION: Dasatinib versus Imatinib Study in Treatment-Naive CML Patients; DDX47: DEAD (Asp-Glu-Ala-Asp) box polypeptide 47; EFS: Event-free survival; ENESTnd: Evaluating Nilotinib Efficacy and Safety in Clinical Trials–Newly Diagnosed Patients; EUTOS: European Treatment and Outcome Study; FOS: FBJ osteosarcoma oncogene; G6PD: Glucose-6-phosphate dehydrogenase; GAB2: GRB2 (growth factor receptor-bound protein 2)-associated binding protein 2; HSP: Heat shock protein; IGSF2: Immunoglobulin superfamily, member 2; IRIS: International Randomized Study of Interferon and STI571; IS: International scale; JUN-B: Protooncogene originally identified as the transforming factor in avian sarcoma virus 17; KD: Kinase domain; LASP1: LIM and SH3 (Src homology region 3) protein 1; LRPPRC: Leucine-rich pentatricopeptide repeat containing; LTB4R: Leukotriene B4 receptor; MEK: Mitogen-activated protein kinase (MAPK) kinase; MMR: Major molecular response; NOB1: NIN1/RPN12 binding protein 1; OCT-1: Organic cation transporter-1; OS: Overall survival; PARP: Poly (ADP-ribose) polymerase; PDK1: Pyruvate dehydrogenase kinase, isozyme 1; PFS: Progression-free survival; Ph+: Philadelphia chromosome–positive; PKC: Protein kinase C; PP2A: Protein phosphatase 2A; PRAME: Preferentially expressed antigen in melanoma; PSMA1: Proteasome subunit, alpha type, 1; RALGDS: Ral guanine nucleotide dissociation stimulator; RB: Retinoblastoma; SCARB1: Scavenger receptor class B, member 1; SLC25A3: Solute carrier family 25, member 3; SMAD1: Portmanteau of *C. elegans* small body size (sma) and *D. melanogaster* mothers against decapentaplegic (MAD); SSBP2α: Single-stranded DNA binding protein 2α; TKI: Tyrosine kinase inhibitor; TRIM24: Tripartite motif containing 24; WNT: Portmanteau of *D. melanogaster* wingless (Wg) and mammalian integration 1 (Int1); XIAP: X-linked inhibitor of apoptosis.

## Competing interests

Conflict of interest disclosures: Dr. Pinilla-Ibarz has received honoraria from Novartis and Bristol-Myers Squibb and research support from Novartis, Bristol-Myers Squibb, and Ariad. Drs. Sweet and Zhang have no conflicts to report.

## Authors’ contributions

LZ and JPI conceived of the topics of discussion. All authors helped draft the manuscript, critically reviewed and revised the manuscript, and read and approved the final manuscript.
